# Preventive aerobic training preserves sympathovagal function and improves DNA repair capacity of peripheral blood mononuclear cells in rats with cardiomyopathy

**DOI:** 10.1038/s41598-022-09361-z

**Published:** 2022-04-19

**Authors:** Paola Victória da Costa Ghignatti, Mariana Kras Borges Russo, Tiago Becker, Temenouga Nikolova Guecheva, Luciele Varaschini Teixeira, Alexandre Machado Lehnen, Maximiliano Isoppo Schaun, Natalia Motta Leguisamo

**Affiliations:** 1 Institute of Cardiology of Rio Grande do Sul/University Foundation of Cardiology (IC/FUC), Av. Princesa Isabel, 370, Porto Alegre, Rio Grande do Sul Brazil; 2 Department of Mechanical Engineering, Federal University of Rio Grande do Sul, Porto Alegre, Rio Grande do Sul Brazil; 3 Institute of Molecular Biology “Roumen Tsanev”, Bulgarian Academy of Sciences, Sofia, Bulgaria

**Keywords:** Cancer, Cardiology

## Abstract

To evaluate the effect of preventive aerobic exercise training on sympathovagal function, cardiac function, and DNA repair capacity in a preclinical model of doxorubicin (DOX)-induced cardiomyopathy. Forty male Wistar-Kyoto rats were allocated into four groups (n = 10/group): D (DOX-treated) and C (controls) remained sedentary, and DT (DOX-trained) and CT (control-trained) performed aerobic training 4 days/week, during 4 weeks before exposure to DOX (4 mg/kg/week during 4 weeks) or saline solution. We evaluated cardiac function (echocardiography), hemodynamic and sympathovagal modulation (artery-femoral cannulation), cardiac troponin T levels, and DNA repair capacity (comet assay). Exercise training preserved ejection fraction (D: − 14.44% vs. DT: − 1.05%, p < 0.001), fractional shortening (D: − 8.96% vs. DT: − 0.27%, p = 0.025) and troponin T levels (D: 6.4 ± 3.6 vs. DT: 2.8 ± 1.7 ng/mL, p = 0.010). DOX increased heart rate variability (C: 27.7 ± 7.9 vs. D: 7.5 ± 2.2 ms^2^, p < 0.001) and induced sympathovagal dysfunction (LF/HF, C: 0.37 ± 0.15 vs. D: 0.15 ± 0.15, p = 0.036) through exacerbation of sympathetic function (LF, C: 0.22 ± 0.01 vs. D: 0.48 ± 0.24 Hz, p = 0.019). Peripheral mononuclear blood cells of DT animals presented lower residual DNA damage (D: 43.4 ± 8.4% vs. DT: 26 ± 3.4%, p = 0.003 after 1 h). Cardioprotective effects of preventive aerobic exercise training are mediated by preservation of sympathovagal function and improvement of DNA repair capacity of peripheral blood mononuclear cells.

## Introduction

Doxorubicin (DOX) is a broad-spectrum antineoplastic agent and the main representative drug of anthracyclines^1^. DOX is largely employed in the treatment of both adult and pediatric hematological and solid malignancies^2^. However, clinical use of DOX is limited due to induction of irreversible damages to off-target tissues, particularly kidneys, liver and heart^3^.

DOX-induced myocardial toxicity (or cardiotoxicity) occurs in 3–26% of the patients who underwent oncological treatment^4^, and it is a potential life-threatening condition that might occur following treatment with anthracyclines-based regimens^5^. Although a consensus clinical definition of cardiotoxicity has not been reached yet, diagnosis and monitoring are based on the studies of left ventricular function by echocardiography^6,7^ and serum markers of cardiac damage, in particular, cardiac troponin T levels^8^.

Cardiac dysfunction following DOX treatment occurs in a dose-dependent manner^9^. However, anthracyclines deleterious effects in the cardiovascular system are not restricted to myocardium as vascular function is also impaired due to a negative modulation of sympathetic and parasympathetic nervous system^10^. Indeed, exposure to anthracyclines are associated with neurohumoral and sympathovagal imbalance^11,12^, which is thought to be a consequence of upregulation of β2-adrenoceptors^13^ and decreased expression of M2 receptors in cardiomyocytes^14^. Hence, increased sympathetic activity contributes, at least in part, to a higher redox state (or oxidative stress) associated with the evolution of a poor cardiovascular prognosis^15,16^. Thus, heart rate variability and sympathovagal dysfunction have been hypothesized as key mechanisms underlying the anthracycline-induced cardiomyopathy and major contributors to the severity and the prognosis of cardiotoxicity^12^.

On the other hand, exercise training is a recognized approach not only for cardioprotection/rehabilitation for adults with cardiovascular diseases, but also for mitigation of cancer-related fatigue of patients with cancer^17^. Also, we conducted a systematic review with meta-analysis on preclinical models in rodents regarding the effects of exercise training in DOX-induced cardiomyopathy^18^. We showed that trained DOX-treated animals improved 7.40% in fractional shortening when compared with sedentary DOX-treated animals and that may be associated with improved autonomic function. Also, the effects of exercise training suggested a greater cardioprotective effect of exercise training prior to DOX exposure.

It is well known that physical training improves autonomic function in humans^19^ and in animal models^20^. More recent evidence has also shown that DOX-induced cardiomyopathy (hemodynamic parameters, baroreflex sensitivity and baroreflex effectiveness index, cardiac autonomic tone, and left ventricular function) were attenuated after resistance training in rats^21^. However, to the best of our knowledge, we found no studies evaluating the effect of aerobic training on autonomic function in rats with DOX-induced cardiomyopathy.

Regarding DNA repair, physical exercise modulates the expression of sirtuins (nicotinamide adenine dinucleotide-dependent deacetylases) in the skeletal muscle, that work as stress adaptors sensing intracellular NAD+ changes^22^. The mammalian sirtuins (SIRT1–SIRT7) are involved in regulation of energy metabolism, antioxidant activity and DNA repair. SIRT1 regulates nucleotide excision repair (NER), homologous recombination (HR) and non-homologous end joining (NHEJ) after genotoxic insult, while SIRT6 stimulates base excision repair (BER) and is also involved in HR and NHEJ^23^. Moreover, SIRT6 has a role as sensor recognizing DNA double-strand breaks, permitting initiation of DNA damage response (DDR)^24^. DDR induction was observed after DNA double-strand breaks recognition by the synergistic action of SIRT1 and SIRT6, allowing DNA repair in human and mouse cells^25^. Association between physical activity and increased DNA repair capacity was shown in humans^26^.

Although sympathovagal disbalance has undeniable influence on the pathogenesis of heart failure irrespective of the etiology^27,28^, and exercise is a well-documented strategy to exert beneficial effects on the sympathetic and parasympathetic nervous system control to the heart^29^, only one preclinical study has investigated these factors conjunctively within the context of anthracycline-induced cardiomyopathy so far^30^. Thus, we aimed to investigate the effects of preventive aerobic exercise training on myocardial and sympathovagal function of rats with DOX-induced cardiomyopathy. Secondly, we also evaluate the DNA repair capacity. We hypothesized that exercise training is able to mitigate the damage induced by DOX on cardiac function, on sympathovagal balance and on the DNA repair capacity, preserving or reducing changes in the analyzed variables, such as ejection fraction, sympathetic and parasympathetic components, and damage to the DNA, among others.

## Results

Four deaths occurred during the experimental observation period in the intervention groups (one in D and three in DT), from which two deaths occurred during cannulation surgery (one in D and one in DT), and two deaths occurred in the 24 h following the surgery (two in DT). No death was exclusively attributed to DOX toxicity. Overall, DOX-treated animals presented lower motor activities, alopecia, chromodacriorea. At gross necropsy examination, we observed organ injury (ascites, chylous ascites, hemorrhagic ascites, ballooning liver, hepatic adherence to adjacent organs, stoned bowel stools and whitish solidified inguinal adipose tissue).

**Figure 1:**
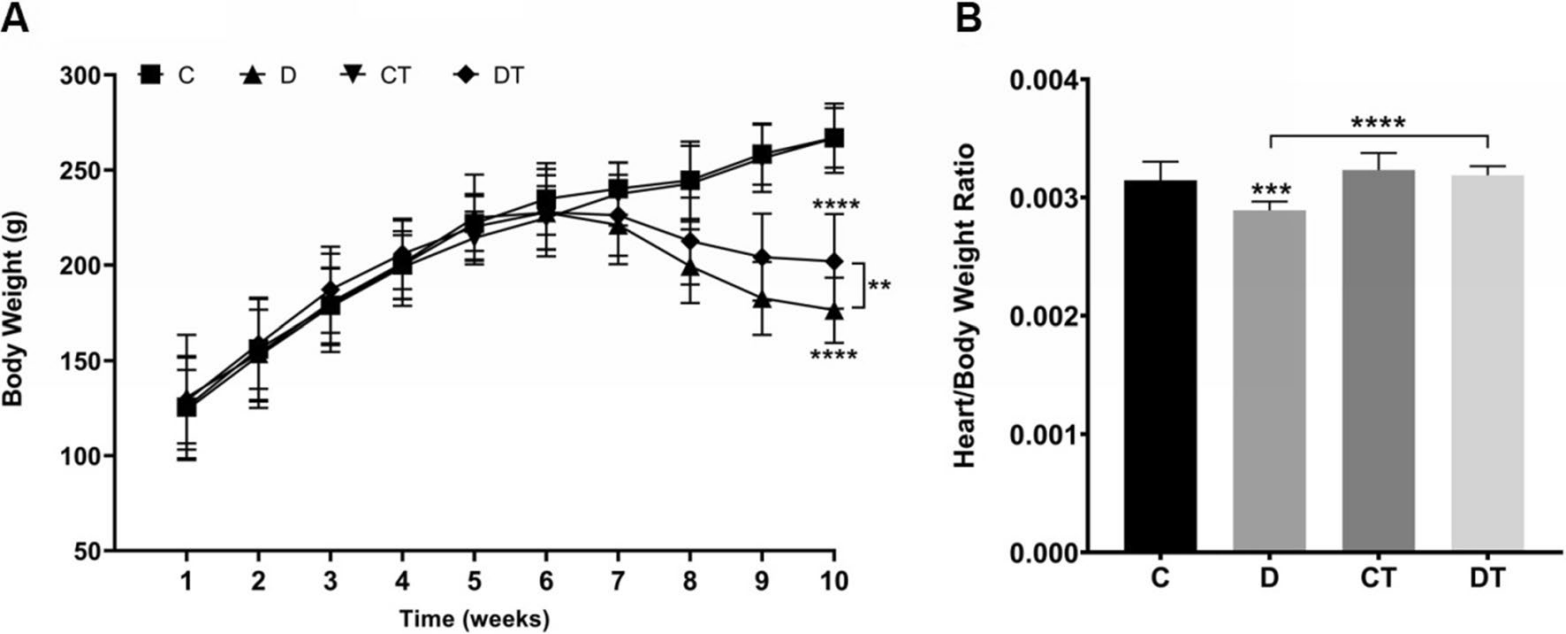
Preventive physical training improves functional capacity and attenuates doxorubicin-induced general toxicity. (**A**) Body weight (g); (**B**) Heart weight/body weight ratio. C: control (n = 10); D: doxorubicin-treated (n = 10); CT: control-trained (n = 10); DT: doxorubicin-trained (n = 10). One or Two-way ANOVA and Tukey’s post-hoc. **p < 0.01, ***p < 0.001, ****p < 0.0001 above error bars in comparison to control group.

**Figure 2:**
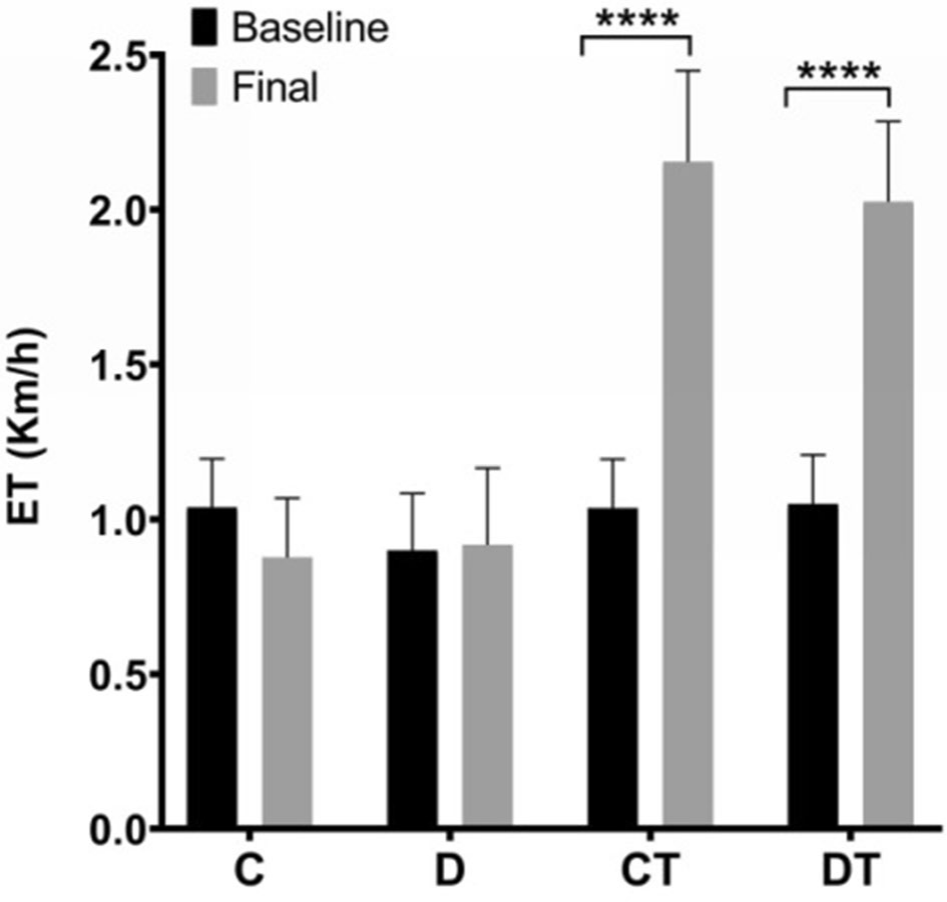
Maximal exercise test (ET). C: control (n = 10); D: doxorubicin-treated (n = 10); CT: control-trained (n = 10); DT: doxorubicin-trained (n = 10). One or Two-way ANOVA and Tukey’s post-hoc. ****p < 0.0001 above error bars in comparison to control group.

Any differences were observed among experimental groups at baseline regarding body weight (Fig. 1A) and, statistically, none of the groups showed differences at baseline (Fig. 2). Also, no changes were observed at each of four weeks of exercise training (before DOX treatment) with regard to body weight (Fig. 1A).

### DOX-induced effects on body weight, cardiac function, and sympathovagal modulation

Both sedentary and trained DOX-treated animals presented an impairment in body weight gain at week 8 (D = 18.77% and DT = 13.06% vs. C, p < 0.001 for both comparisons)—Fig. 1A. In addition, post-mortem examination revealed that sedentary DOX-treated animals presented a mean reduction of 34.08% in heart/body weight ratio (p < 0.001)—Fig. 1B.

**Figure 3:**
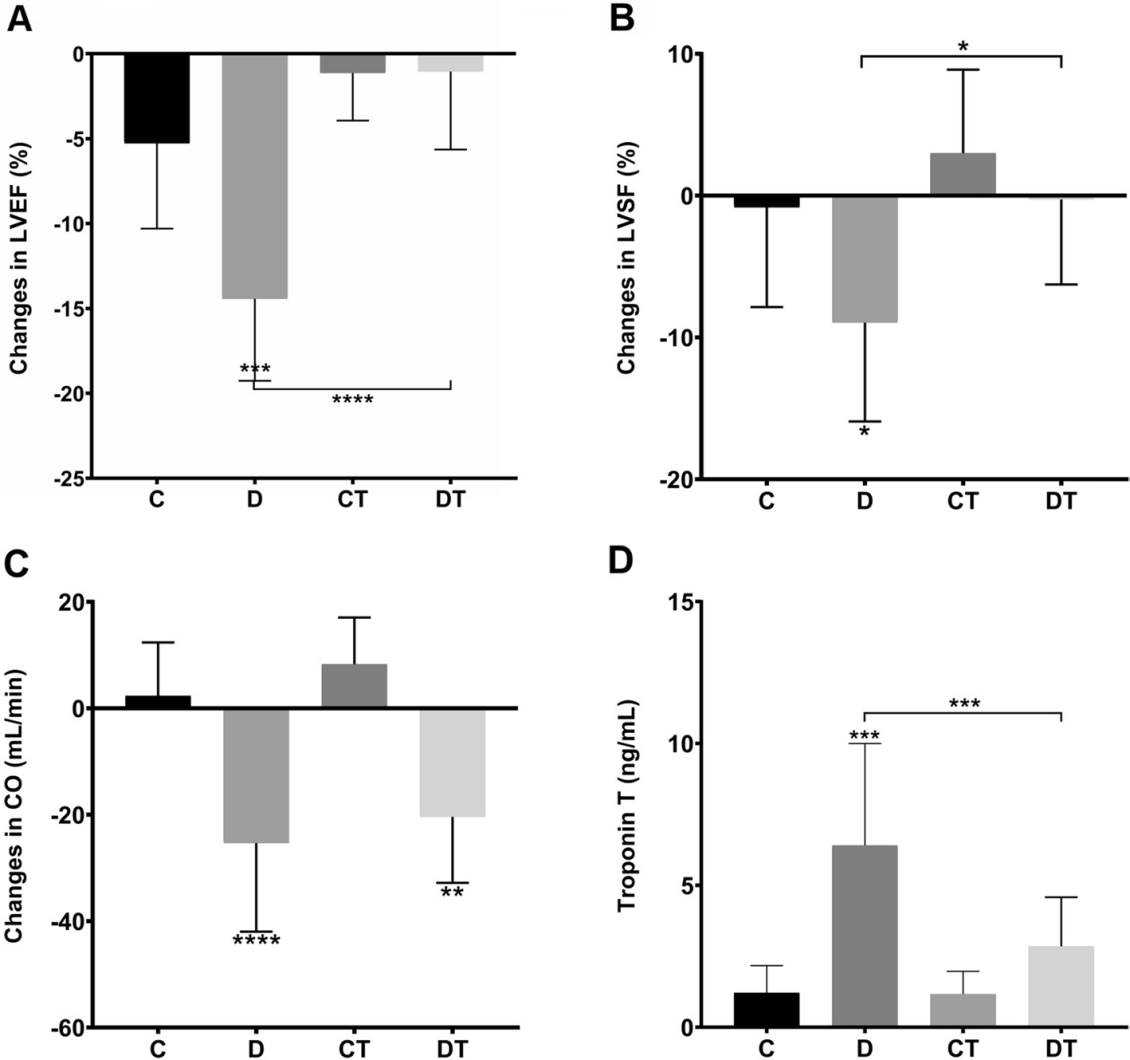
Preventive aerobic exercise training preserved cardiac function of doxorubicin-treated animals. (**A**) Mean changes in left ventricle ejection fraction (LVEF) and (**B**) shortening fractional (LVSF) from baseline to final assessment (%);(**C**) Mean changes in cardiac output (CO, mL/min); (**D**) Cardiac Troponin T (ng/mL). C: control (n = 10; for Troponin T: n = 8); D: doxorubicin-treated (n = 10; for Troponin T: n = 9); CT: control-trained (n = 10; for Troponin T: n = 7); DT: doxorubicin-trained (n = 10; for Troponin T: n = 8). One-way ANOVA and Tukey’s post-hoc. *p < 0.05, **p < 0.01, ***p < 0.001, ****p < 0.0001 above error bars in comparison to control group.

Regarding cardiac function, DOX cumulative dose of 16 mg/kg induced a marked reduction in LVEF, LVFS and cardiac output with a mean change of − 14.44% (p < 0.001), − 8.96% (p = 0.387) and − 25.35 mL/min (p < 0.001) from baseline, respectively (Fig. 3A–C). In addition, the cardiac damage marker cTnT was in consonance with echocardiographic results as sedentary DOX-treated animals presented higher levels (C = 1.22 ± 0.96 ng/mL vs. D = 6.41 ± 3.59 ng/mL, p < 0.001), Fig. 3D. Representative images of cardiac echocardiography are shown in Fig. 1-Sup (“Supplementary Material”).

**Figure 4:**
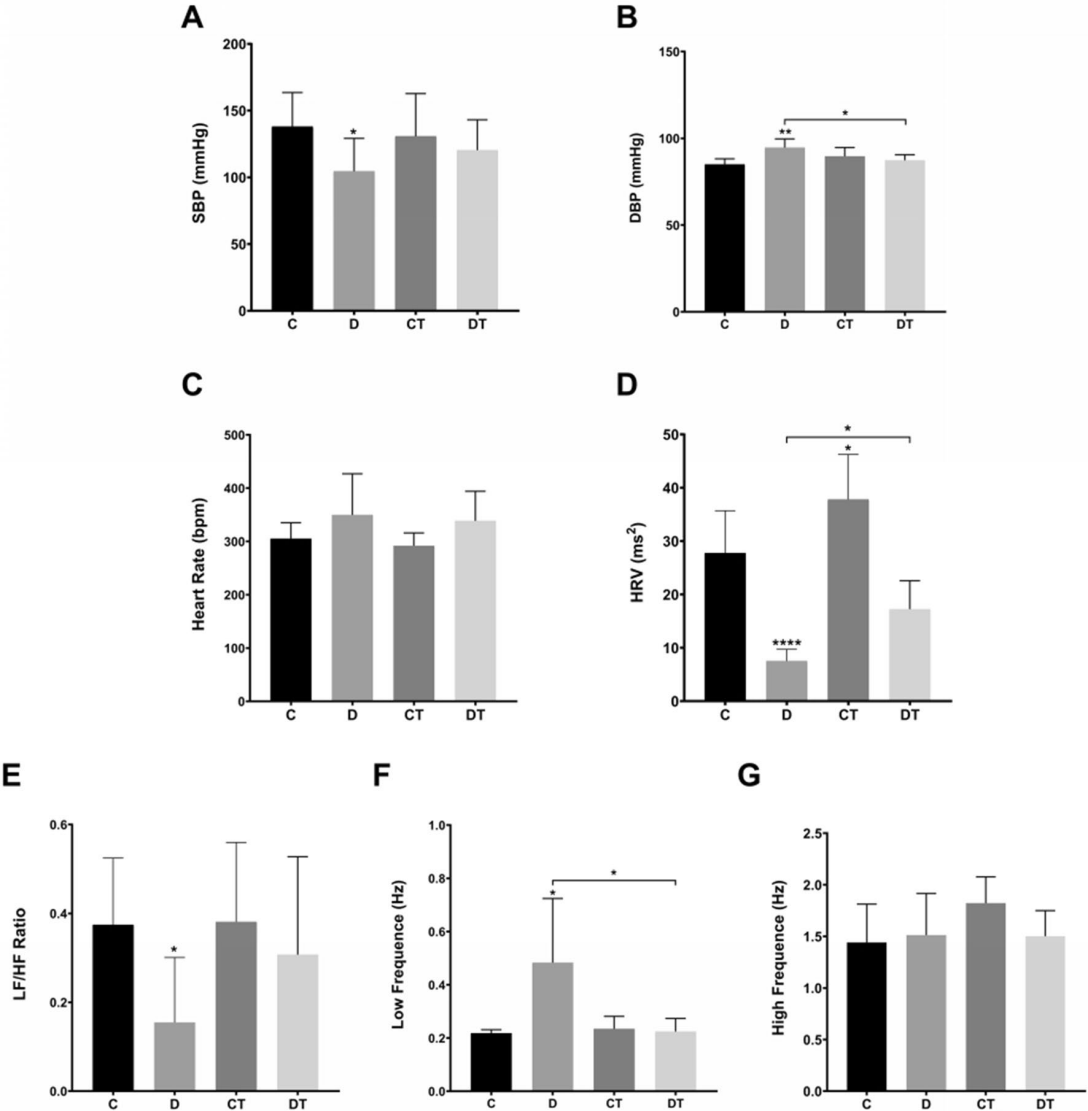
Preventive exercise training attenuates hemodynamic and sympathovagal imbalance induced by doxorubicin. (**A**) SBP: systolic blood pressure (mmHg); (**B**) DBP: diastolic blood pressure (mmHg); (**C**) Heart rate (bpm); (**D**) HRV: heart rate variability (ms^2^); (**E**) LF/HF ratio: low frequency/high frequency ratio as sympathovagal balance; (**F**) LF: low frequency representative of sympathetic component (Hz); (**G**) HF: high frequency representative of parasympathetic component (Hz). C: control (for hemodynamic parameters: n = 8; for sympathovagal parameters: n = 5); D: doxorubicin-treated (n = 8); CT: control-trained (for hemodynamic parameters: n = 10; for sympathovagal parameters: n = 7); DT: doxorubicin-trained (for hemodynamic parameters: n = 7; for sympathovagal parameters: n = 5). One-way ANOVA and Tukey’s post-hoc. *p < 0.05, **p < 0.01 above error bars in comparison to control group.

Hemodynamic and sympathovagal evaluations are presented on Fig. 4. DOX treatment reduced SBP (C = 138.03 ± 22.55 mmHg vs. D = 104.58 ± 24.63 mmHg, p = 0.019) (Fig. 4A) and increased DBP levels (C = 85.07 ± 3.13 mmHg vs. D = 94.71 ± 4.97 mmHg, p = 0.004) (Fig. 4B). Although DOX treatment did not change the heart rate (Fig. 4C), it strongly reduced HRV (C = 27.75 ± 7.95 ms^2^ vs. D = 7.52 ± 2.19 ms^2^, p < 0.001) (Fig. 4D). Furthermore, DOX treatment caused an imbalance between sympathetic and parasympathetic nervous system components (C = 0.37 ± 0.15 vs. D = 0.15 ± 0.15, p = 0.036) (Fig. 4E), with a particular exacerbation of sympathetic function (C = 0.22 ± 0.01 Hz vs. D = 0.48 ± 0.24 Hz, p = 0.019) (Fig. 4F). Individual analysis of sympathovagal system components revealed that DOX exposure decreased sympathetic (Fig. 4F) but not parasympathetic (Fig. 4G) modulation.

### Exercise-induced effects

**Figure 5:**
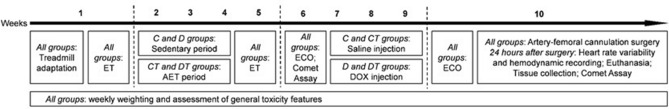
Experimental design. C: control (n = 10); D: doxorubicin-treated (n = 10); CT: control-trained (n = 10); DT: doxorubicin-trained (n = 10); AET: aerobic exercise training period; ECO: echocardiographic assessment; ET: maximal exercise test.

The preconditioning aerobic training protocol improved the cardiorespiratory capacity of all trained animals (Fig. 5B). CT and DT groups showed an increase maximum running speed in the ET, when compared with those that remained sedentary for 4 weeks prior to DOX or saline intervention (C = 0.88 ± 0.19 km/h vs. CT = 2.15 ± 0.29 km/h; D = 0.92 ± 0.25 km/h vs. DT = 2.02 ± 0.26 km/h; p < 0.001).

Regarding body weight behavior, exercised DOX-treated animals showed superior mean body weight gain in comparison to sedentary DOX-treated animals (D = 183.57 ± 19.03 g vs. DT = 204.19 ± 22.90 g, p = 0.028) since week 9 (Fig. 1A), mitigating the effect of DOX on body weight. Moreover, post-mortem examination revealed that sedentary DOX-treated animals presented a mean reduction of 34.08% in heart/body weight ratio (p < 0.001), but exercise training maintained this parameter similar to controls (Fig. 1B).

In turn, LVEF and LVFS, but not cardiac output, were preserved in animals that underwent to aerobic training prior to DOX exposure—Fig. 3. DT group animals showed a mean change of − 1.05% of LVEF (p < 0.001) and of − 0.27% of LVFS (p = 0.025) from baseline, which collectively indicates that exercise training preserved the cardiac function of DOX-treated animals. Also, exercised DOX-treated animals did not present evidence of cardiac damage considering cTnT (DT = 2.85 ± 1.73 ng/mL vs. D, p = 0.010)—Fig. 3D.

Figure 4 shows hemodynamic and sympathovagal imbalance features. Hemodynamical benefits of exercise training were identified regarding only to DBP (p = 0.044). Futhermore, while exercise training increased HRV in exercised control group (CT = 37.82 ± 8.42 ms^2^ vs. C, p = 0.048), in DOX-treated animals exercise training have only attenuated HRV decrease (DT = 17.24 ± 5.32 ms^2^ vs. D, p = 0.044). Exercise training prevented the reduction of sympathetic modulation in DOX treated animals (DT = 0.22 ± 0.05 Hz vs. D, p = 0.022), but exerted no effect in parasympathetic component—Fig. 4F,G.

### Preventive exercise training and DNA repair capacity

**Figure 6:**
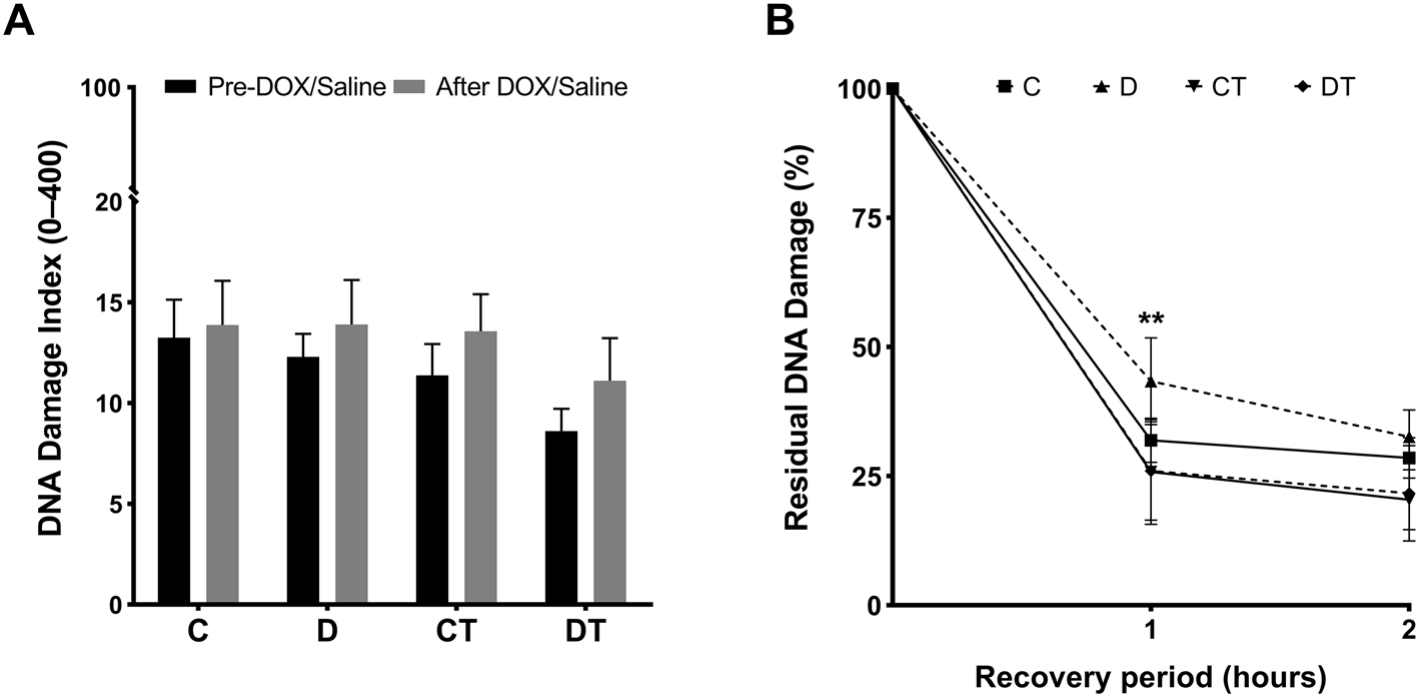
DNA damage and repair capacity in peripheral blood mononuclear cells of doxorubicin-treated animals. (**A**) Basal DNA damage evaluated in Comet Assay; (**B**) DNA repair capacity in blood cells after ex vivo treatment with tert-butyl hydroperoxide, followed by a repair period for 1 h or 2 h in the absence of the oxidizing agent. C: control (n = 8); D: doxorubicin-treated (n = 10); CT: control-trained (n = 8); DT: doxorubicin-trained (n = 8). Two-way ANOVA and Bonferroni’s post-hoc, standard error mean. **p < 0.01 above error bars in comparison to doxorubicin-trained group at same post-incubation period.

The basal DNA damage in blood mononuclear cells does not differ between the trained and sedentary groups before and after the treatment with DOX (Fig. 6A). This is an expected result as comet assay detects recent DNA damage. The DOX-induced DNA damage is repaired in few hours or the highly damaged cells are eliminated by apoptosis. The ex-vivo treatment of blood mononuclear cells with the oxidizing agent TBHP led to the formation of breaks in the DNA. After 1 and 2 h of repair, the reduction of damaged DNA was greater in the blood cells of DOX-treated trained rats (DT) in relation to the DOX-treated (D) (D = 43.4 ± 8.4% vs. DT = 26 ± 3.4%, p = 0.003 after 1 h), indicating induction of DNA repair by exercise training (Fig. 6B).

## Discussion

The main findings of this study were (1) DOX (cumulative dose of 16 mg/kg) induced myocardial damage, reduced left ventricle function, decreased HRV and sympathovagal balance and overactivated sympathetic drive one week after treatment ending; (2) 4-week preventive aerobic training preserved global cardiac function and protected from DOX-induced sympathovagal dysfunction, as it attenuated HRV decrease and maintained normal LF/HF due to preservation of parasympathetic activity, and (3) improved DNA repair capacity. Thus, we accept the hypothesis that exercise training is able to mitigate the damage induced by DOX on cardiac function, on sympathovagal balance and on the DNA repair capacity.

The impairment of optimal doses of antineoplastic therapies or even discontinuation due to development of cardiotoxicity are the most concerning drawbacks during oncological treatments^31^. Despite unquestionable advances in antineoplastic agents and survival rates improvements, the high antitumor effectiveness of anthracyclines maintains these drugs as the backbone of several chemotherapy regimens^32,33^. Therefore, it is crucial to identify cardioprotective strategies which do not hamper (or yet potentialize) the cytotoxic effects of already proven effective antineoplastic therapies.

In this regard, the beneficial effects of regular physical activity on heart function in pathological conditions are well documented^34^. Indeed, exercise is strongly associated with positive regulation of antioxidant^35^ and anti-apoptotic activities and enhancement of mitochondrial function and calcium transport, which are all impaired by DOX^36^. Besides, these direct effects on myocardium, exercise-mediated benefits on cardiac function also reflect the improvements on sympathetic and parasympathetic nervous system. Exercise training improves baroreflex control of heart rate due to increased vagal activity^37^. Also, since moderate to high intensity aerobic and strength exercises are recommended concomitantly to oncological treatment to counteract loss of cardiac function and improve cardiorespiratory fitness and oncological outcomes^38,39^, it has been proposed as an adjuvant cancer treatment.

Although exercise prescription during the chemotherapy treatment has been reported to improve physical performance, muscle strength and aerobic capacity in humans^40^, to reach the appropriated level of adherence or compliance to exercise may be a challenge due to the many side effects of treatments^41^. A study exploring the predictors of adherence to an 18-week supervised aerobic and strength exercise program during breast cancer treatment reported that modality of oncological treatment (radiotherapy + chemotherapy), high body mass index (BMI), high physical fatigue level, low peak oxygen consumption at baseline predicted low attendance^42^. Hence, while the preconditioning might bypass some of these obstacles (fatigue, exposure to radiotherapy), in the same time it might also work to mitigate others, such as decrease of BMI and increase of aerobic capacity prior to oncological treatment.

Our group conducted a systematic review with meta-analysis that gathered information available in the literature on the influence of regular physical exercise on the prevention and/or attenuation of DOX-induced cardiotoxicity in rodents^18^. Ghignatti et al.^18^ show that physical exercise is cardioprotective in the scenario of DOX-induced cardiotoxicity by preserving cardiac function (measured by the fraction of shortening of the left ventricle). Furthermore, they concluded the superiority of aerobic modalities, preferring supervised and routine activities over unsupervised and sporadic ones. However, regarding the intensity and duration of physical exercise, even those of low intensity can induce favorable metabolic adaptations^43^. Thus, according to our research, we developed the current training protocol prior to DOX, with aerobic characteristics, of moderate intensity and supervised.

As several chemotherapy agents, anthracyclines also promote cachexia, sarcopenia and cardiac atrophy in cancer patients, mostly due to oxidative stress, disruption of protein synthesis/degradation balance and lipid metabolism and reduction of glucose uptake in myocardial, skeletal muscle and adipose tissues^44,45^. Clinical manifestations include increased general toxicity, shortness of breath, lethargy, reduced exercise tolerance and cardiac dysfunction^46^. We observed marked impairment of weight gain and cardiac atrophy in sedentary animals receiving a cumulative dose of 16 mg/kg of DOX, which were all significantly attenuated or prevented in rats from trained DOX-treated group. Our results add to the current body of evidences the importance of preconditioning aerobic training as a non-pharmacological strategy to prevent DOX-induced skeletal muscle loss of mass, weakness and fatigue^47^.

Additionally, DOX-treated animals presented a mean difference of − 8.1% LVEF, which consistently reproduces a largely reported fact in cancer survivors who had undergone anthracyclines-based treatments^48^. Reduced left ventricular mass may decline within 6 months after initiating anthracycline-based therapies due to myocellular atrophy and/or cardiomyocyte loss, resulting in inappropriate ventricular remodeling^48,49^. This is particularly relevant on the context of exercise prescription for those with a cancer diagnosis since loss of cardiac mass is strongly related to reduced functional capacity and intolerance to exercise^50^. It is likely that physiological heart hypertrophy secondary to physical exercise^51^ explain why heart weight/body weight was not different between exercised and control animals^52^.

Most of the guidelines and clinical trials define cardiotoxicity as a ≥ 10% drop in LVEF from baseline to a value ≤ 50% by echocardiography^33^. However, the threshold of clinically important left ventricle dysfunction and how is it measured are still on debate. Furthermore, the assessment of classical biomarkers of myocardial damage have been extensively investigated to detect early manifestations of cardiotoxicity^53^. In particular, even mild increases of cardiac troponins are associated with systolic dysfunction in patients treated with cardiotoxic cancer therapy^8^. Our results show that a week after receiving 16 mg/kg of DOX, the sedentary animals presented a mean reduction of 14.44% in LVEF and 8.96% in LVFS and a mean increase of 425.41% in cTnT in comparison to controls, which were all completely hindered in trained DOX-treated animals, as reported elsewhere^54–56^. It is important to highlight that our group conducted a systematic review with meta-analysis of preclinical studies assessing the effects of exercise training on DOX-induced cardiomyopathy^18^, and the vast majority report LVSF as main outcome of cardiac function rather than LVEF. Although this choice might lie on the assumption that fractional shortening represents a global analysis of any diastolic dimension lost in systole and reflects LVEF, it is only accurate if left ventricle contracts homogeneously^57,58^.

It has been also postulated that myocardial deleterious effects of chemotherapy agents, in particular anthracyclines, may affect cardiac sympathetic and parasympathetic functions and, therefore, contribute to the development and the severity of cardiac complications during and after oncological treatment^59^. In fact, monitoring of heart rate and blood pressure variabilities have been proposed as diagnostic markers of early and subclinical cardiotoxicity^29^. Spectral analysis of HRV reflects neurogenic control on HR and quantifies the activity of sympathetic (low frequency, LF) and parasympathetic components (high frequency, HF). Thus, the LF/HF ratio represents the sympathovagal balance to the heart^60^.

This is the first study to address the cardiac sympathovagal function in exercised rats with early onset of DOX-induced cardiomyopathy. By the end of the observational period, while the sedentary DOX-treated animals showed significantly decreased HRV, LF/HF and increased sympathetic activity, the exercised DOX-treated animals kept these parameters equal to controls. The literature on the role of the sympathovagal control of cardiac function in preclinical models of DOX-induced cardiomyopathy is scarce and highly heterogeneous, mostly because of the variability of protocols for cardiotoxicity induction (cumulative dose fractioning, schedules, etc.) and timing of assessment of cardiac function (early or late onset). Contrarily to our findings, it has been previously reported that DOX increases HRV in rats due to sympathetic over parasympathetic dominance^61^. Although this study assessed sympathovagal function 7 days after the last dose of DOX, the animal model consisted in colorectal tumor-bearing rats receiving a cumulative dose of only 4.5 mg/kg. Also, despite the use of a similar protocol for DOX-induced cardiomyopathy (cumulative dose 15 mg/kg) by two other studies, spectral analyses of cardiac sympathovagal function were performed 35 and 70 days following the last DOX injection. Lončar-Turukalo, et al.^12^ reported increased HRV and alterations in LF/HF with increased sympathetic contribution after 35 days, which was lately confirmed by^62^. Also, Vasić’s study reported that LF and LF/HF returned to normal levels after 70-days post DOX treatment. Our animal model reproduces the clinical presentation of acute cardiotoxicity^63,64^, which is the occurrence of myocardial damage during or soon after therapy and is characterized by mitochondrial dysfunction, lipoperoxidation, cellular membrane instability and necrotic cell death^65,66^. Since DOX treatment upregulates adrenergic β2 receptors and, consequently responsiveness to sympathetic stimulation, only 35 days after the last injection^13^, we suggest that decrease of HRV in DOX-treated animals might reflect that myocardial adaptation have not been fully reached yet.

Regular physical activity is associated with reduced sympathetic tone and with stimulation of vagal dominance^67,68^. The sympathovagal components of trained animals remained equal to controls, which might indicate an exercise-mediated neurogenic protection. However, the single study supporting the effects of preventive exercise training effects counteracting DOX-induced reduction of sympathetic drive and preserving normal HRV^30^. Although we have also identified upregulation of sympathetic component in sedentary DOX-treated animals, Moguilevski et al. exercise protocol is entirely different from ours as they used rabbits receiving 1 mg/kg twice weekly of DOX for 4 or 6 weeks (cumulative dose of DOX 8 or 6 mg/kg) after a single treadmill session 12 m/min for 2 min^30^.

So far, there is no available clinical evidence concomitantly addressing the effects of antineoplastic treatments and exercise training on sympathovagal balance and HRV in cancer patients. Survivors of childhood acute lymphoblastic leukemia treated with DOX presented significant reduction in the sympathovagal nervous system in the long term^59^. Also, patients at high risk of developing cardiotoxicity (according to the cumulative dose of DOX) have more pronounced sympathovagal disbalance due to sympathetic component exacerbation and reduction of vagal dominance and HRV^59^. Indeed, low HRV has been considered a marker of cardiac dysfunction associated with poor overall cardiovascular health and unfavorable prognosis^69^. On the other hand, regular exercise training is a notorious cardioprotective practice to improve vagal tone and increase HRV while simultaneously suppress sympathetic activity in the heart of individuals under pathological conditions, such as congestive heart failure or diabetes mellitus^70^. The repetitive activation of the sympathetic nervous system during each physical exercise session results in attenuation of sympathetic dominance^71^. In counterpart, in order to conserve and store energy for the basal functions of the body, parasympathetic predominance is stimulated^72^. Taken together, these evidences emphasize the relevance of developing future preclinical studies not only to extend the comprehension of chemotherapy-induced modulation of cardiac sympathovagal nervous system within the pathogenesis of cardiotoxicity, but also to investigate how the cardioprotective strategies might influence on other aspects of cardiac disfunction rather than in irreversible changes of left ventricle function.

Chemo- and radiotherapy have side effects due to extensive damage to healthy cells. In these cells, the cytotoxic effect of both modalities induces DNA damage and cell death, leading to endothelial dysfunction and subsequent inflammation, culminating in senescence, apoptosis, thrombogenesis, mitochondrial dysfunction and fibrosis, all of which promote cardiovascular disease^73^. The cytotoxicity of DOX involves the formation of DNA breaks and the generation of reactive oxygen species. Decreased repair capacity in association with induction of plasma superoxide dismutase was reported in breast cancer patients treated with DOX, indicating involvement of oxidative stress^74^. Our exercise training protocol increased the capacity for DNA repair in DOX treated rats in the present study, which can result in cardioprotective effect. Physical exercise imposed metabolic demand leads to increased activity of the AMP-activated protein kinase (AMPK), which regulates glucose and lipid homeostasis maintaining the cell energy status. Consequent activation of SIRT1 promotes deacetylation of key proteins, such as tumor suppressor protein p53 and 8-oxoguanine DNA glycosylase-1 (OGG1), playing an important role in the adaptive response to physical training^75^. The p53 can act as regulator of DNA integrity and cellular homeostasis, promoting cell cycle arrest and DNA repair after induction of low to moderate level of DNA damage, or cell death by apoptosis after extensive damage. On the other hand, the increased hydrogen peroxide production in mitochondria during the exercise activates p53, which in turn modulates oxidative metabolism, mitochondrial biogenesis, autophagy and mitophagy. Its action provides protection of mitochondrial DNA and increase in ATP production by transcriptional induction of Electron Transport Chain (ETC) proteins^76^. OGG1 plays a role in the repair of 8-oxoguanine (the main oxidative lesion into DNA), and in regulation of gene expression. SIRT1 can also decrease the inflammation, modulating the redox-sensitive nuclear factor kappa B (NF-*κ*B), and enhance the expression of the antioxidant enzymes Catalase and Superoxide dismutase^77,78^. Exercise training increased the levels of SIRT1, SIRT3 and the anti-apoptotic Bcl-2 in the heart, while the level of the proapoptotic protein Bax decreased^79^. Moreover, exercise training in humans can act on the body increasing the DNA repair capacity and antioxidant responses^26^. In this way, it can counteract the excessive formation of oxidative damage resulting from the action of DOX and promote an efficient adaptive response. Physical exercise in patients with chronic obstructive pulmonary disease also led to a significant decrease in lipid peroxidation in blood plasma, decreased susceptibility to exogenous mutagens and improved efficiency of DNA repair^80^.

In conclusion, our study adds new evidence on the significance of sympathovagal modulation as a key-component of DOX-induced cardiotoxicity. Global cardiac function was preserved in those animals who underwent regular moderate-intensity aerobic training prior to DOX exposure. We suggest that these protective effects occurred due to exercise-mediated influence on both central and peripheral autonomic systems, mostly through induction of vagal dominance and control of sympathetic drive. Moreover, the improvement of DNA repair efficiency in peripheral blood mononuclear cells of trained animals could indicate an increase of systemic repair capacity that has possibly contributed to counteract DOX-induced oxidative injuries and DNA lesions in cardiomyocytes, resulting in cell survival. Future preclinical studies still have to address the effects of different exercise modalities and training variables (such as duration, intensity, frequency and volume) and to define the safest and effective protocol to add value in the setting of cancer adjuvant therapeutics.

Limitations of the study: this study did not include tumor-bearing animals to evaluate the effects of exercise on DOX-induced cardiomyopathy. Further studies still have to address this feature along with animal models reproducing the clinical features of most cancer patients (older age, menopause, comorbidities, etc.) and the effects of detraining on (1) chemotherapy-induced loss of muscular strength, cachexia and fatigue, and (2) the acquired cardioprotective effects in preconditioning period regarding global cardiac function.

## Methods

### Animals and experimental design

This study was approved by Comitê de Ética para Uso de Animais (Porto Alegre, Rio Grande do Sul) under protocol UP5517/18, and all experiments were performed in accordance to Sociedade Brasileira para Ciência de Animais de Laboratório SBCAL/COBEA Alsos, the Animal Research: Reporting of In Vivo Experiments (ARRIVE) guideline was used for the elaboration of this manuscript.

Forty 8-weeks old male Wistar Kyoto rats were kept in animal boxes (4 rats per cage) and maintained under conventional animal facility conditions, with controlled temperature, light and dark cycle (12 h) and access to water and food (Nuvilab CR1, Brazil) ad libitum. Sample size calculation was performed in WinPepi software, version 11.65^81^. In order to detect a mean difference of 4.2% in LVEF between animals that DOX-treated animals exercised or not, a power of 90% and type I error probability of 0.05, sample size was calculated as ten animals per group.

Wistar-Kyoto rats were randomly assigned into four groups (n = 10 rats/group). Control (C), which received 0.9% NaCl intraperitoneally injection once a week during 4 weeks; DOX-treated (D) were intraperitone injected with 4 mg/kg of DOX (Doxorubicin hydrochloride, Fauldoxo^®^, Libbs, São Paulo, Brazil) once a week during 4 weeks (cumulative dose of 16 mg/kg^82–84^), and both groups remained sedentary for 4 weeks before the injections. Control-trained (CT) and DOX-trained (DT) underwent to a 4-week period, 4 times a week aerobic training before receiving 0.9% NaCl or DOX as previously described, respectively.

The animals were weighted weekly and echocardiographic measures were performed 48 h after completing the training protocol or sedentary period and one week after the cumulative DOX dose was reached. Subsequently, also 1 week after concluded the DOX protocol, the animals were submitted to an artery-femoral cannulation surgery. After 24 h, the animals were lightly anesthetized with isoflurane and euthanized by decapitation. Experimental design and temporal outcome assessment are depicted in Fig. 5.

### Maximal exercise test (ET) and exercise training

Prior to the exericse training protocol and at the end, maximum effort capacity was measured by the exercise test^85^. After an adaptation period (3 days) on the treadmill at a speed of 0.3 km/h (15 min), the maximal exercise test was then performed individually at an initial speed of 0.3 km/h with increments of 0.3 km/h every 3 min until voluntary exhaustion of the animal.

Exercise training consisted of a four-weeks moderate-intensity aerobic treadmill training program four times a week^85^, at 0% grade, before DOX or saline administration. The adopted mean intensity was 50% to 80% of the maximum speed evaluated according to ET, with a progressive increment of time. In the first week, the animals started with 25 min at 30–60% intensity and reached 40 min at 40–65% intensity. In the second week, the animals started with 45 min at 40–70% intensity and reached 50 min at 40–75% intensity. In the third week, the animals started with 55 min at 40–75% intensity and reached 55 min at 45–80% intensity. In the fourth week, the animals trained until one hour at 45–80%. Sedentary animals underwent to treadmill adaptation and ET assessments for comparison between groups, and then remained confined in their cages for the same period of exercise training.

### Doxorubicin-induced cardiomyopathy induction

Following the exercise training or sedentary period, the animals were injected intraperitoneally with saline solution (NaCl 0.9%) or 4 mg/kg of DOX (Doxorubicin hydrochloride, Fauldoxo^®^, Libbs, São Paulo, Brazil) once per week during four weeks, reaching a cumulative dose of 16 mg/kg. During that time, the animals were observed for symptoms of pain and received veterinary follow-up when necessary. General toxicity was assessed by qualitative searching for classical features previously described, such as lethargy, irregular breathing, weight loss, fur loss, appearance of coat and tearing/porphyrin.

### Assessment of cardiac function

Echocardiographic (bidimensional and M mode) were performed in two moments: 48 h after the end of physical training protocol or sedentary period, and one week after the fourth DOX or saline injection. The animals were anesthetized with a mixture of 2–3% of isoflurane (100%, 1 mL/mL, Isoforine, Cristália) in 100% oxygen and placed in left lateral decubitus. The echocardiographic analyses were carried out by one blinded examinator in the EnVisor (Philips, Andover, USA) echocardiograph with a 12 MHz transducer. Conventional measurements were obtained from gray scale M-mode images at the level of the papillary muscles and included left ventricular ejection fraction (LVEF, %) and left ventricular fractional shortening (LVFS, %) as previously described^86^.

Additionally, cardiac troponin T (cTnT) levels were assessed at the end of the protocol. The centrifugated plasma from cardiac blood puncture samples from the animals were available to be analyzed for cTnT. The concentrations of cTnT were measured by an immunoassay (Elecsys, STAT; Roche Diagnostics) at the Laboratory of Clinical Analysis of the Institute of Cardiology (RS, Brazil). The reason for choosing troponin T was because its elevation persisted for up to 14 days, which rendered irreversible myocardial injury^87^.

### Assessment of heart rate variability and spectral analysis

One week after the last DOX or saline injection (and 24 h after the last cardiac echocardiography), the animals were anesthetized with a mixture of 2–3% of isoflurane (100%, 1 mL/mL, Isoforine, Cristália) in 100% oxygen and submitted to artery-femoral cannulation surgery, as described by^62^. Briefly, the animal was positioned for surgical insertion of a heparin-filled (10% in sterile saline) polyethylene catheter (PE-10, Biocorp Australia, Huntingdale, Victoria, Australia) in the right femoral artery. The catheter was tunneled subcutaneously for the exit between scapulae. The animals received Tramadol (12 mg/kg intraperitoneally) analgesia after surgery and every 8 h until euthanasia.

Twenty-four hours after artery-femoral cannulation surgery, the catheter was connected to a pressure transducer extension (Strain-Gauge, Narco Biosystem, Miniature Pulse Transducer PR-155; Houston, TX, USA) with an amplifier (Pressure Amplifier HP 8805C, USA) for continuous recording of pulsatile blood pressure for a period of 25 min (after stabilizing the record). All records were performed in conscious rats in a quiet and temperature-controlled (22–24 °C) room to reduce ambient stress and always with the same two observers.

The analogical signal was digitally converted by a data acquisition and recorder system using WinDaq Data Acquisition Software (DATAQ Instruments Inc., Akron, Ohio, USA). The mean of the pressure values recorded by the wave peaks is equivalent to the systolic blood pressure (SBP), while the mean of the pressure values recorded by the wave valleys is equivalent to the diastolic blood pressure (DBP).

Heart rate (HR), heart rate variability (HRV), sympathetic (low frequency, LF) and parasympathetic (high frequency, HF) components of autonomic nervous system were obtained from blood pressure recordings. Sampling rates of continuous segments of 300 overlapping beats allowed a posterior off-line evaluation of HRV, in time domain (ms^2^) using the fast Fourier transform in MATLAB software, version 9.7 (Math Works Inc., Massachusetts, USA). The LF and HF components were also expressed in hertz (Hz) for spectral analysis of each component. The LF/HF was calculated as the ratio of the absolute values of LF and HF components.

### Euthanasia and tissue collection

Total blood was collected by cardiac puncture and was centrifugated to separate plasma. Immediately before euthanasia, the animals were anesthetized with isoflurane until they manifest no resistance. Next, the animals were euthanized by decapitation for tissue collection. A median laparotomy was performed, allowing access to the heart by insertion into the diaphragm. The hearts were collected, sanitized in saline and individually weighted to calculate heart weight/body weight ratio^88,89^ to infer cardiac enlargement of each animal.

### DNA damage and repair capacity in comet assay

DNA damage was analyzed by alkaline comet assay and repair capacity in peripheral blood mononuclear cells was evaluated as described in Agnoletto et al.^74^ with modifications. In summary, peripheral blood was collected in anticoagulant EDTA tubes from the animals’ tail after completing the training protocol and from the heart by cardiac puncture at the time of euthanasia. The 500 µL fraction of blood was added to 2.5 mL of Ficoll Histopaque^®^-1077 (Sigma-Aldrich) in a 15 mL Falcon tube and then centrifuged at 400×*g* for 30 min. The mononuclear cells ring was washed in phosphate buffered saline (PBS) and centrifuged at 200×*g* for 10 min. The supernatant was discarded and the pellet resuspended in the remaining volume. Agarose pre-coated microscope slides received a mixture of 15 µl sample and 90 µL low melting point agarose at 37 °C. After solidification at 4 °C for 3 min, the negative control slide was immersed in a cold lysis solution at 4 °C for 24 h. For evaluation of DNA repair capacity, the cells were exposed to the oxidizing agent tert-butyl hydroperoxide (TBHP) [100 µM] for 5 min at 4 °C. After that the slides were washed in PBS and the slides for evaluation of the initial DNA damage (zero post-incubation time) were added to the lysis solution. The remaining slides were incubated for 1 or 2 h in RPMI culture medium with 10% fetal bovine serum at 37 °C to permit DNA repair and then immersed in cold lysis solution.

After overnight lysis, the slides were transferred to alkaline solution (300 mM NaOH, 1 mM EDTA, pH > 13) for 30 min. Electrophoresis was performed at 300 mA and 25 V (0.94 V/cm) for 30 min. The slides were then neutralized with 10 mM Tris buffer (pH 7.5), stained with silver nitrate and analyzed at 200× magnification using an optical microscope. The damage index (0–400) is calculated from one hundred randomly selected cells per animal that were visually scored according to the tail length and intensity in five classes (from undamaged, 0 to maximally damaged, 4). The residual DNA damage after 1 h and 2 h of post-incubation (allowing repair) was calculated as per cent of the initial DNA damage induced by the TBHP treatment (zero time).

### Statistical analyses

Statistical analyses were performed in GraphPad Prism software version 8.0.1 (San Diego, California, USA). The results were presented as mean ± SD (standard deviation) or mean ± SEM (standard error mean). Comparisons between experimental groups and moments were assessed by one or two-way ANOVA, followed by Tukey's or Bonferroni’s post-hoc tests. Statistical significance was set at p < 0.05.

## Supplementary Information

Supplementary Legends.

Supplementary Figure S1.
