# Supplementary material for: Preventive aerobic training preserves sympathovagal function and improves DNA repair capacity of peripheral blood mononuclear cells in rats with cardiomyopathy
Source: Sci Rep. 2022 Apr 19;12:6422. doi: 10.1038/s41598-022-09361-z (PMC9018832; doi:10.1038/s41598-022-09361-z)
Supplement: Supplementary file 1 — Supplementary Legends. [file 41598_2022_9361_MOESM1_ESM.docx]

**Figure 1-Sup (supplementary material). Representative images of cardiac echocardiography.** C: control; D: doxorubicin-treated; CT: control-trained; DT: doxorubicin-trained.
