# Supplementary figures and images for: Preventive aerobic training preserves sympathovagal function and improves DNA repair capacity of peripheral blood mononuclear cells in rats with cardiomyopathy
Source: Sci Rep. 2022 Apr 19;12:6422. doi: 10.1038/s41598-022-09361-z (PMC9018832; doi:10.1038/s41598-022-09361-z)

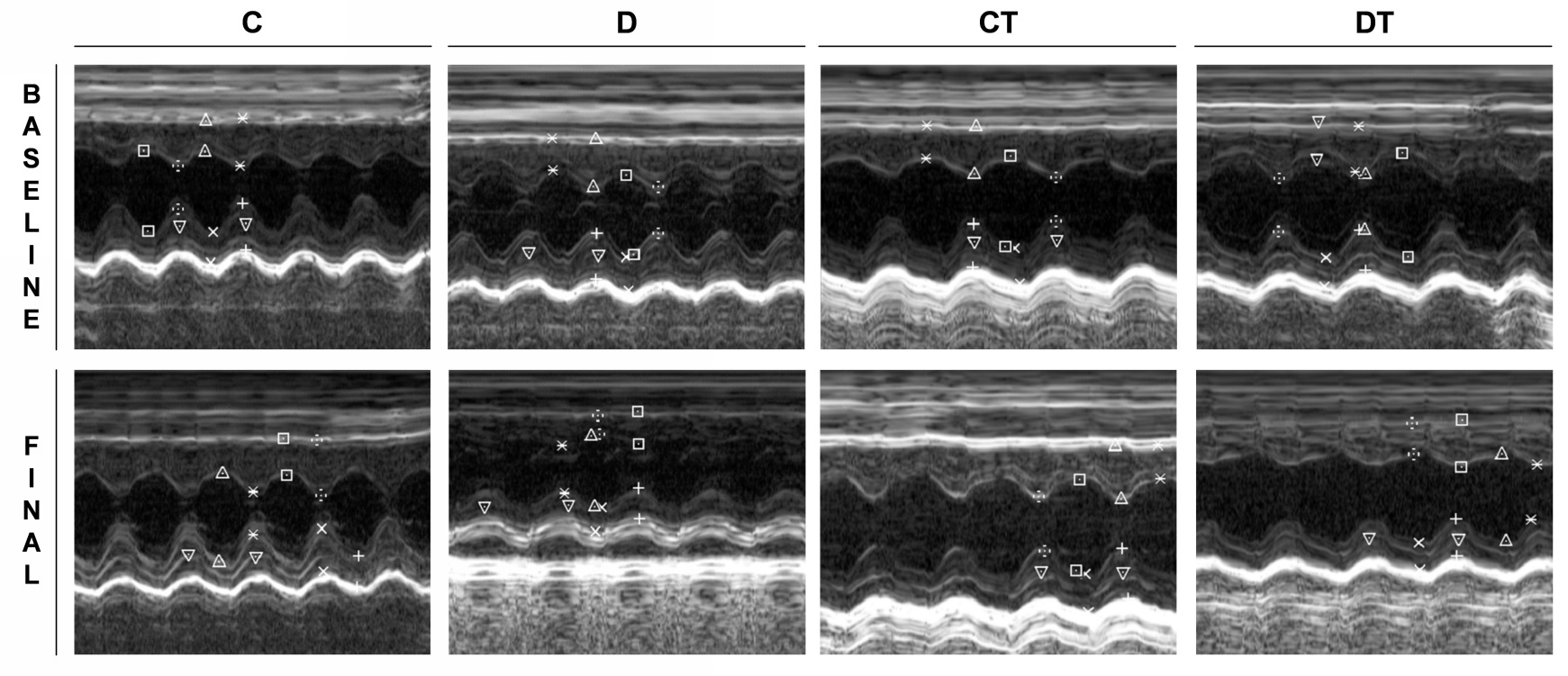

Supplement: Supplementary file 2 — Supplementary Figure S1. [file 41598_2022_9361_MOESM2_ESM.jpg]
